# Anorexia nervosa: from purgative behaviour to nephropathy. a case report

**DOI:** 10.1186/1757-1626-2-46

**Published:** 2009-01-13

**Authors:** Emilia Manzato, Maria Mazzullo, Malvina Gualandi, Tatiana Zanetti, Giovanni Scanelli

**Affiliations:** 1 Centre for Eating Disorders, Azienda Ospedaliero Universitaria di Ferrara "Arcispedale Sant'Anna", Corso Giovecca 203, 44100 Ferrara, Italy; 2 Department of Internal Medicine, Azienda Ospedaliero Universitaria di Ferrara "Arcispedale Sant'Anna", Corso Giovecca 203 44100 Ferrara, Italy

## Abstract

**Background:**

Individuals who suffer from Anorexia Nervosa refuse to maintain a minimally normal body weight, are intensely afraid of gaining weight and exhibit a significant disturbance in the perception of the shape and size of their body. Postmenarchal females with this disorder are amenorrohic. In the Binge-Eating/Purging subtype individuals regularly engage in binge eating and purging behaviour (i.e self-induced vomiting or misuse of laxatives, diuretics, or enemas).

Hypokalaemia is often seen in chronic Anorexia Nervosa, especially that of the purging type (ANp), and, as well as electrocardiographic anomalies, this can lead to tubulointerstitial nephritis (hypokalaemic nephropathy) with typical histological characteristics. The physiopathological mechanisms behind this damage are linked to altered stimulation of vasoactive mediators, and to the ammonium-mediated activation of the alternative complement pathway. However, it has not yet been ascertained whether a variant of the pathway specific for ANp [1], exists.

**Case presentation:**

We describe herein a case of hypokalaemic nephropathy in a patient affected by chronic ANp who presented to our Centre for Eating Disorders.

**Conclusion:**

Hypokalaemia can provoke cardiovascular alterations as well as muscular and renal complications, and thus potential renal damage needs to be investigated in patients suffering from long-term purgative anorexia.

## Case presentation

The patient, a 33-year-old female Caucasian nurse, was referred to our Centre for Eating Disorders by her GP. She was affected by a severe untreated form of ANp with a duration of illness of 19 years. She smoked 15 cigarettes per day for over 15 years and drank 1 glass of wine at meals.

Four years previously, the patient had been kept under observation by the Psychiatry department for attempted suicide by medicinal overdose. The patient had also been treated with anti-depressives for about 15 years, although this therapy had been suspended about three months before her referral to our Centre.

No significant pathologies emerged in the review of the medical history of the patient's family. During the psychological assessment she reported the separation of her parents when she was a child and her difficulty in maintaining intimate relationships in adulthood. She embarked on her first diet following the death of her maternal grandmother; at this time she considered her weigh excessive (BMI 25). Three years prior to her arrival at the Centre, she had undergone a cone biopsy for adenocarcinoma in situ and had suffered a spontaneous abortion in the 3rd month of pregnancy.

She presented the main core eating disorder symptoms: she denied her underweight condition, she was obsessed with thoughts of food, weight and shape, she thought she couldn't stop bingeing and vomiting. She had decreased energy and difficulties in organizing her daily life. She worked compulsively and reported difficulties to relax. Affective disregulation and problems in her interpersonal relationships emerged in her clinical picture.

Upon her arrival at the Centre, she complained asthenia, lipothymia, ectopic heartbeat and abdominal pain; the intestine was not assessable due chronic laxative abuse. Binging (3–4 times/day) and the following severe purgative practices were disclosed: self-induced vomiting (28 episodes/week), laxative intake (1 cp day of senna) and diuretic use (occasional). Daily physical hyperactivity was also divulged: 2 hours of running or workout.

The diagnostic assessment revealed ANp and the concomitant Axis II Condition: Borderline Personality Disorder (according to the Diagnostic and Statistical Manual of Mental Disorders, Edition IV-TR) [2].

The following data were revealed: weight 49 kg, height 1.67 m, BMI 17.5 kg/m^2^; blood pressure 125/85 mmHg; heart rate 72/min; respiratory rate 32/min; cutaneous temperature 36,2°C. Almost all of the teeth underwent odontoiatric cures or were substituted. Hair, nails and skin appeared normal.

Laboratory findings: serum total calcium 2.33 mmol/l, sodium 140 mEq/l, plasmatic proteins 7,6 g/dl with albumin 54,9% (4,17 g/dl). Hypokalaemia (3.1 mEq/l) was found and responded only to prolonged endovenous potassium (30 mEq/day of potassium chloride for 3 weeks), despite the patient's claimed cessation of the aforementioned purgative activity. Due to the persistent hypokalaemia, renin and aldosterone levels were evaluated, and the following data emerged: secondary hyperaldosteronism (Aldosterone > 100 ng/dl, active renin 408.9 μU/ml); creatinine in the normal range (0.8 mg/dl) and reduced creatinine clearance (70 ml/min); urinary electrolytes and ammonia were not available because the patient left the hospital against medical advice. Haemogas analysis in ambient air revealed pO_2 _127.80 mmHg, pCO_2 _38.8 mmHg, pH 7.39, HCO_3- _22.80 mmol/l, and SO_2 _98.80%.

Oesophagogastroduodenoscopy (OGDS) showed grade "A" reflux oesophagitis (according to Los Angeles Classification [3]); abdominal ultrasound revealed bilateral reduction of the thickness of the renal cortex; renal scintigraphy exposed a bilateral delay in the arrival phase of the trace and no signs of obstructive uropathy were noted.

## Discussion

Individuals with Anorexia nervosa binge eating/purging type lapse in their resticting patterns, they binge eat and subsequently adopt vomiting and/or laxative abuse as compensatory behaviours.

ANp can induce hypokalaemia via the loss of water and salts (diuretic and laxative abuse), metabolic alkalosis (loss of acids through vomiting) and hyperaldosteronism, secondary to hypovolaemia.

Prolonged hypokalaemia leads to a distinct clinical variety of chronic tubulointerstitial nephritis, hypokalaemic nephropathy, which is characterised by tubular atrophy, mononucleocyte infiltration into the interstitial spaces, interstitial fibrosis and juxtaglomerular hyperplasia. Three mechanisms lead to its onset: increased ammoniogenesis, activation of vasoactive mediators and arterial hypersensitivity to Na_+_.

Ammonium produced in the kidney acts as a urinary buffer; for each molecule of NH_4+ _excreted in the urine, one bicarbonate ion is returned to the circulation and this replaces the HCO_3- _lost during titolation of the non-volatile acid. Hypokalaemia stimulates HCO_3- _reabsorption via an as yet undescribed mechanism. Reabsorption of HCO_3- _in the proximal tubule is coupled with activation of the Na_+_-H_+ _antiport (Na_+ _reabsorption, H_+ _secretion), thus, for each HCO_3- _reabsorbed, one H_+ _is excreted. This event may stimulate ammoniogenesis while there is a sufficient amount of NH_3 _to buffer the H_+ _secreted. Increased ammoniogenesis in the renal cortex during hypokalaemia leads to the amination of C_3_, with consequent activation of the alternative complement pathway and deposition of protein in the tubule.

Furthermore, hypokalaemia causes alterations in the levels of vasoactive mediators: an increase in vasoconstrictor stimuli (ACE, ET-1 and subtype B α-adrenergic receptors) and a reduction in vasodilatory stimuli (EDRF-1 and PGE_2_). This explains the ischaemic lesion pattern in the kidneys and the principal location of these lesions in the medulla, which is more sensitive to low pO_2 _[4,5].

Moreover, hypokalaemia is associated with increased activity of the co-transporter Na_+_K_+_2Cl_- _in the ascending limb of the loop of Henle, with consequent hydrosaline retention and elevated arterial pressure (AP); AP, which is highly sensitive to Na_+_, has been found to be elevated even after resolution of hypokalaemia, which demonstrates that both alterations in vasoactive mediator levels and irreversible tubular lesions occur [6].

It is also reasonable to hypothesize that different mechanisms cooperate in the genesis of hypokalaemia. In a patient affected by chronic AN with serum K_+ _of 2 mmol/l despite the administration of exogenous potassium, Luthra [7] hypothesised the presence of a tubular lesion able to diminish reabsorption and increase secretion of potassium, promoted by the secondary hyperaldosteronism typical of these forms.

It is possible that a type of hypokalaemic nephritis specific to Anorexia Nervosa exists. Reported data [1,8-10] demonstrate that, in ANp patients with severe, prolonged hypokalaemia, irreversible renal damage may result; after correction of the ion imbalance in fact, the markers of renal function remain altered and renal biopsy reveals several, or sometimes all, of the histological characteristics described. The more prolonged the hypokalaemia and the more severe the nephropathy, the higher the probability that End Stage Renal Disease (ESRD) will develop.

The relative importance of these pathophysiological mechanisms in determining the clinical picture is still controversial; moreover, in the present case we don't know the serum levels of ammonium; furthermore, the haemogas analysis shows, instead of a metabolic alkalosis, a metabolic acidosis, against which the patient answers by increasing the respiratory rate; again, we don't know the histological damage (we did not perform a renal biopsy).

Nevertheless, on the basis of the aforementioned mechanisms and of the available literature, we can hypothesize that ANp may, in the most severe and prolonged cases, lead to a permanent renal lesion of double aetiology: 1) hypovolaemia, which causes ischaemic lesion directly; and 2) hypokalaemia, which causes ischaemic lesion indirectly, via ammonium-mediated damage.

## Abbreviations

Anp: Anorexia Nervosa, binge/purging type; BMI: Body Mass Index; OGDS: Oesophagogastroduodenoscopy; ACE: Angiotensin Converting Enzyme; ET-1: Endothelin-1; EDRF-1: Endothelium-Derived Relaxing Factor-1; PGE2: Prostaglandin E2; AP: Arterial Pressure; ESRD: End Stage Renal Disease

## Consent

Informed written consent was obtained from the patient for publication of this case report and accompanying images. A copy of the written consent is available for review by the Editor-in-Chief of this journal.

## Competing interests

The authors declare that they have no competing interests.

## Authors' contributions

EM and TZ analysed and interpreted the patient data regarding the psychiatric disease; MM, MG and GS analysed and interpreted the data pertaining the renal disease; EM, MM and GS were the major contributors in writing the manuscript. All authors read and approved the final manuscript.
